# Phase Evolution in Mn_1.6_Zn_0.2_Ni_0.6_Mg_0.2_Al_0.4_O_4_ High-Entropy Oxide Films by Heat Treatment

**DOI:** 10.3390/ma17235967

**Published:** 2024-12-05

**Authors:** Wei Ren, Xianhai Liu, Wenting Wu, Weili Wang

**Affiliations:** 1 School of Physical Science and Technology, Northwestern Polytechnical University, Xi’an 710072, China; renwei@xupt.edu.cn; 2 School of Science, Xi’an University of Posts & Telecommunications, Xi’an 710121, China; liuxianh_link@163.com; 3 North West Electric Power Design Institute, Xi’an 710075, China; wuwenting@nwepdi.com

**Keywords:** high-entropy oxide, spinel oxide, thermistor, anneal, conductive behavior

## Abstract

In this work, Mn-Zn-Ni-Mg-Al multi-layer films were annealed in air at different temperatures to form spinel-structured Mn_1.6_Zn_0.2_Ni_0.6_Mg_0.2_Al_0.4_O_4_ high-entropy oxide films. X-ray diffraction results demonstrate that the films possess a polycrystalline spinel phase as well as impurity phases: when annealed at 650 °C and 750 °C, MnO_2_ and Al_2_O_3_ impurity phases exist; at 950 °C, an Al_2_O_3_ impurity phase exists. Only at 850 °C does a pure spinel phase exist. However, the film at 750 °C exhibits the best conductive behavior, which indicates that the impurity phases may not have to be removed to maintain the best electrical properties of the film.

## 1. Introduction

Manganese-based spinel oxides are classical negative temperature coefficient (NTC) materials for thermistors and thermal detectors [1,2,3,4]. It is well known that Mn_3_O_4_, with a formula of AB_2_O_4_ (A: divalent Mn^2+^ at the oxygen tetrahedral lattice interstitial, B: trivalent Mn^3+^ at the oxygen octahedral lattice interstitial), is the prototype material of these oxides. Manipulation of the mechanical, optical, and electrical properties of Mn_3_O_4_ used to be accomplished through introducing heterogeneous metal cations (e.g., Ni^2+^, Co^3+^) [5,6,7]. The temperature-dependent conductive behavior of Mn-based spinel oxides could be ascribed to the directional polaron hopping between Mn^3+^ and its adjacent lattice-distortion-induced Mn^4+^ at the octahedron interstitial sites [8,9], while this behavior may be further affected by the partial replacement of Mn cations by alien divalent or trivalent cations. However, the conductive performance of these oxides was found to deteriorate seriously with time (i.e., aging issue) due to cationic migration, which greatly limits their application in industry [5].

Recently, the idea of designing high-entropy alloys (mixing five or more metals with similar molar ratios to form a multiple-principal-element alloy with a single-phase-like FCC or BCC structure) has been disseminated to Mn-based spinel oxides, which are called high-entropy oxides (HEOs, i.e., mixing five or more divalent and/or trivalent cations in oxides). Theoretically, the purpose of designing HEOs is to induce the sluggish diffusion effect among the cations, reduce the cationic migration, and correspondingly alleviate the aging issue [6,7,8]. This is because the sluggish diffusion of high-entropy alloys leads to the decrease in diffusion rates with the increase in configurational entropy (i.e., the increase in the alloying elemental number) [9,10]. Experimentally, the sluggish diffusion effect has been confirmed in Mn_(1.9−x)_Zn_0.2_Ni_0.6_Mg_x_Al_0.3_O_4_ (0 < x < 1) HEOs [8], although the chemical bonding and cationic distribution in HEOs are quite different to those in high-entropy alloys. The effects of thermal dynamic behaviors of multiple cations on the conductive properties of Mn-based HEOs are still not clear. In particular, the solubility of some constitutive alien cations may be very limited in Mn-based HEOs, which do not allow the existence of a single equi-cationic spinel phase (the contents of multiple cations are similar). Therefore, the precipitation phases may be generated and the conductive behavior of the HEOs altered.

In this study, the electron beam evaporation (EBE) method was used to deposit Mn-Zn-Ni-Mg-Al multi-layer metallic thin films on Si substrates, and post-annealing in air followed to prepare Mn_1.6_Zn_0.2_Ni_0.6_Mg_0.2_Al_0.4_O_4_ (MZNMAO) HEO films. The effects of the annealing temperature on the microstructure and electrical properties of MZNMAO films were explored.

## 2. Materials and Methods

### 2.1. Sample Preparation

In order to prepare MZNMAO films, a single-crystalline Si wafer (650 μm thick) was cut into small pieces as the substrates with a dimension of ~1.5 × 1.5 cm^2^. Then, they were dipped into an alcohol solution, an acetone solution, as well as deionized water for ultrasonic cleaning for 15 min. After that, the Si substrate pieces were taken out of the deionized water and blown dry with an earball. Immediately after that, they were adhered to the sample holder with high-temperature adhesive tape. Mn, Zn, Ni, Mg, and Al metal particles (99.99% purity) were, respectively, put into 5 designated copper crucibles. Next, the working conditions of the EBE system (the vacuum level, the power of electron gun…) and the process parameters of the films (the film thickness, the substrate temperature, and the deposition rate) were set up. The substrate temperature was set to 300 °C, and the pressure in the EBE chamber was pumped down to below 5 × 10^−3^ Pa. In order to pre-melt the material, the spot size of the electron beam was manually adjusted and the spot position was varied by observing the film thickness and deposition rate through a crystal oscillator, and the films were deposited on the Si substrate pieces according to the molar ratio of Mn:Zn:Ni:Mg:Al = 4:1:3:1:2 (nominally, the film thickness ratio) with a total film thickness of 150 nm. The obtained multi-layer metallic films were, respectively, annealed in a furnace at the temperatures of 650, 750, 850, and 950 °C for 60 min, thereby obtaining MZNMAO HEO films, which were labeled as S1, S2, S3, and S4.

The careful selection of film parameters facilitated the deposition of high-quality multi-layer materials. Specifically, a metal multi-layer with six sub-layers, i.e., Mn, Zn, Ni, Mg, Al, and Mn, was specially designed and sequentially deposited on Si substrates. Among the multi-layer, a Mn sub-layer with half thickness was deposited on the top-most layer, since Mn is easily oxidized to form manganese oxide and oxygen is capable of diffusing through the manganese oxide layer into the other metal sub-layers below. In addition, because of the high melting points, a Ni sub-layer and another half of a Mn sub-layer were placed as the middle and bottom-most sub-layers, respectively, while low-melting-point Zn, Mg, and Al layers were placed between Ni and the top-most/bottom-most Mn sub-layers. Another reason for putting Mn as the bottom-most sub-layer was to prevent inter-diffusion between metal sub-layers and substrate: when the Zn, Mg, and Al sub-layers started to melt with the annealing temperature rising, they would diffuse more easily into the Mn and Ni sub-layers, thus reducing the possibility of these elements diffusing into the silicon substrate.

**Figure 1:**
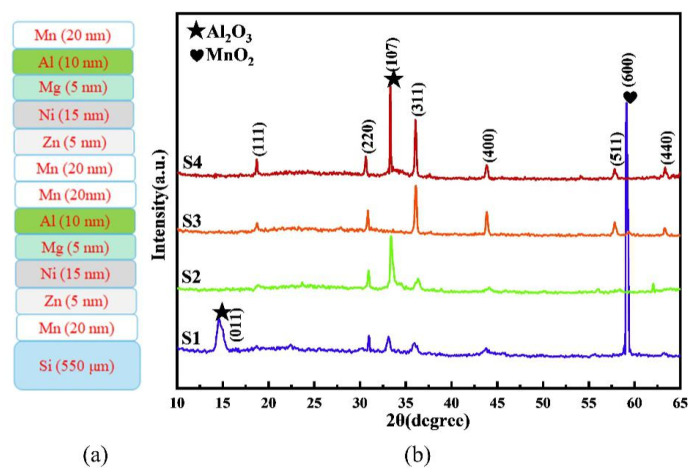
(**a**) Schematic of an untreated multi-layer metal film and (**b**) XRD patterns of the MZNMAO films with different annealing temperatures.

In order to further improve the inter-diffusion of metal sub-layers, two deposition cycles were set up. Each cycle included six sub-layers, i.e., there were twelve sub-layers in total. The thickness of the multi-layer film was set to 150 nm. The molar ratio of different metal elements could be expressed by the following equation:

(1) $$n=\frac{m}{M}=\frac{\rho v}{M}=\frac{\rho AH}{M}$$

where *n* was the mole number, *M* the molar mass of the metal, *ρ* the density of the metal, *A* the area of the Si substrate, and *H* the film thickness. Replacing the known density and molar mass of five different metals into Equation (1), the film thickness ratio of different elements was found to be roughly equal to the molar ratio of these elements. Based on the multi-layer thickness of 150 nm and Equation (1), the thickness values of each metal sub-layer in each deposition cycle were 20 nm for the top-most Mn sub-layer, 5 nm for the Zn sub-layer, 15 nm for the Ni sub-layer, 5 nm for the Mg sub-layer, 10 nm for the Al sub-layer, and 20 nm for the bottom-most Mn sub-layer. The schematic of the metal multi-layer film is shown in Figure 1a. The deposition rate of each sub-layer was kept at 0.05 nm/s. The filament current of the electron gun was set at 90 mA. Under the precise control of the coating sequence, film thickness, and deposition time, inter-diffusion between metal sub-layers was obtained with desirable structural properties.

### 2.2. Characterization Method

The structural phases of annealed samples were identified using Cu Kα radiation X-ray diffraction (XRD, 6100SAS, Shimadzu Corporation, Kyoto, Japan). Raman spectra were measured using a Raman spectrometer (FI532, Beijing Zhuo Li Han Guang Instrument Co., Beijing, China) in a backscattering configuration. A scanning electron microscope (SEM, JSM-7610F Plus, Japan JEOL Ltd., Tokyo, Japan) was utilized to scan the surface and cross-section morphology of the films to observe the morphological changes in the films. X-ray photoelectron spectroscopy (XPS, ThermoScientific™K-Alpha™, Suzhou Sainz Instrument Co., Suzhou, China) was tested to check the chemical states of the elements in the samples. In order to obtain the room-temperature (*R*-*T*) curves of the samples, a mask with round dot-shaped holes of about 1 mm in diameter covered the sample surface for the deposition of gold electrodes on the film surface. Afterwards, the samples were annealed in a furnace at 200 °C for half an hour to obtain ohmic contact between the gold electrode and film. Then, the two-probe method was used for the electrical measurements of the films under the vacuum in a commercial *R*-*T* test system (LNT-800, Wuhan Jiayitong Technology Co., Wuhan, China).

## 3. Results and Discussion

Figure 1a shows the coating order of the metal multi-layer and the thickness of each sub-layer. The XRD patterns of the MZNMAO films after the thermal treatment of the multi-layer with annealing temperatures from 650 to 950 °C are shown in Figure 1b. Although the nominal composition of the films (Mn_1.6_Zn_0.2_Ni_0.6_Mg_0.2_Al_0.4_O_4_) is complicated, six representative diffraction peaks, (111), (220), (311), (400), (511), and (440), are observed for S1~S4 films, indicating that the spinel phase has formed. In particular, the S3 film at 850 °C exhibits a unanimous polycrystalline spinel structure with the cubic Fd3m space group. At the lower temperatures (S1 and S2) or higher temperature (S4), the impurity phases of MnO_2_ and Al_2_O_3_ can be observed.

For the main spinel phase of S1~S4 films, the intensities of (111), (220), (311), (440), and (511) diffraction peaks gradually increase with the annealing temperature. This trend is especially apparent for the strongest orientation peak of (311). As a contrast, the intensities of (400) diffraction peaks firstly increase at the temperature of 650 up to 850 °C, and then decrease at 950 °C. This phenomenon means that the (311) orientation peak is the preferred one for the spinel phase when the annealing temperature increases.

The average grain size of the (311) peaks was calculated according to Scherrer’s formula as follows:

D = kλ/Bcosθ(2)

From Figure 1b, the diffraction peaks are also observed to shift towards the lower diffraction angle, indicating that the lattice constants are dependent on the annealing temperature. The lattice constants were then calculated from the Bragg equation as follows:

*nλ* = 2*d*sin*θ* (3)

where *n* is the number of diffraction levels, *λ* the wavelength of X-rays, *d* the distance between neighboring crystalline planes, and *θ* half of the diffraction angle (i.e., 2*θ*). From the *d* value, the lattice constants of the S1~S4 films could be calculated to be 8.281, 8.884, 8.248, and 8.252 nm.

In addition, several impurity peaks are present. For the S1 film, a strong and sharp (600) MnO_2_ peak and two (011) and (107) Al_2_O_3_ peaks suggest that the diffusion of oxygen is not complete yet: the top-most Mn sub-layer should have been well oxidized, but the lower Al sub-layer just starts to be oxidized (the peak widths of Al_2_O_3_ are wide). A peak from Zn or Mg does not emerge, probably because their contents are relatively low. More importantly, their melting points (419 °C for Zn and 649 °C for Mg) are lower than the annealing temperature of 650 °C and both metal sub-layers have been melted, diffused into the other metal sub-layers, and completely oxidized. As for Al, its melting point is about 660 °C, which is slightly higher than the annealing temperature, suggesting that a metallic Al sub-layer should exist, if without the oxygen diffusion inside. The Al does not quickly diffuse into the other sub-layers, but is oxidized, leading to the emergence of Al_2_O_3_ peaks. As for the Ni sub-layer, Ni diffuses fast and is mixable with Mn and other metallic elements. Therefore, the mixed Mn-Ni-Zn-Mg-O with a little bit of Al cation starts to form the spinel phase (the spinel peaks are weak in intensity and broad in peak width).

When the annealing temperature increases to 750 °C, the MnO_2_ peak and Al_2_O_3_ (011) peak disappear, while the Al_2_O_3_ (107) peak greatly increases in its intensity. This suggests that the MnO_2_ phase has completely transformed into the spinel phase due to the further diffusion of Ni, Zn, and Mg cations as well as the partial diffusion of Al cations. Due to the higher annealing temperature, the crystalline quality of the residual Al_2_O_3_ phase is improved. Similarly, the crystalline quality of the spinel phase is improved, too.

Interestingly, when the temperature rises to 850 °C, the S3 film exhibits a single-spinel phase and no impurity phase forms. The spinel diffraction peaks start to be stronger and sharper, with the (311) orientation as the preferred one. This phenomenon indicates that the MZNMAO HEO film under 850 °C annealing may realize the uniform distribution of Mn, Zn, Ni, Mg, and Al cations inside the film.

When the temperature rises to 950 °C, several spinel peaks of S4 film are relatively stable in intensity, and several slightly increase in their intensities. However, the Al_2_O_3_ (107) peak comes out again: its intensity is even higher and its peak width is sharper. The re-emerging Al_2_O_3_ peak indicates that the Al_2_O_3_ phase precipitates due to the further oxidation of the MZNMAO HEO film. Under a higher annealing temperature, some metallic cations with low chemical valence states, such as Mn^2+^, Mn^3+^, and Ni^+^, are further oxidized to higher chemical valence states, such as Mn^3+^, Mn^4+^, and Ni^2+^, leading to the redistribution of the metallic cations at 850 °C annealing. To meet the neutral electrical charge of the spinel lattice, the Al_2_O_3_ phase precipitates because more Mn^3+^ and Mn^4+^ cations occupy the octahedral sites due to further oxidation. This explains the change in the Al_2_O_3_ (107) peak from 850 to 950 °C.

**Figure 2:**
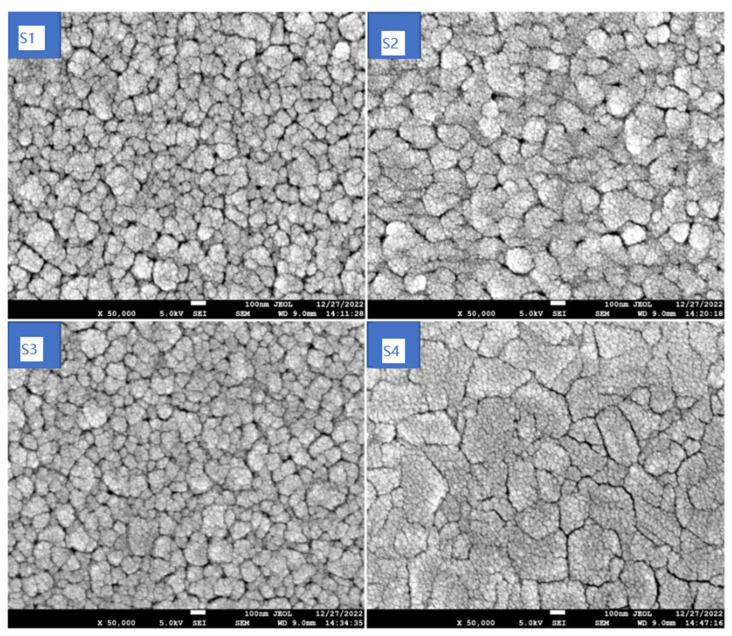
The SEM images of the MZNMAO films with different annealing temperatures: S1 for 650 °C, S2 for 750 °C, S3 for 850 °C, and S4 for 950 °C.

In order to explore the change in the surface morphology with the annealing temperature, SEM analysis was performed on the MZNMAO films. The results are shown in Figure 2. The surface morphologies of the S1–S3 samples are similarly filled with aggregated clusters which are composed of many fine particles. On the S1 surface, the particle size is not uniform, and there are many deep grooves and holes along the boundary of the clusters, resulting in a poor densification of the film surface. On the S2 surface, the particle size is relatively uniform, and the number of holes and grooves is reduced. The S3 surface is more densely packed: the small particles merge into large particles, the particles are in close proximity to each other, the grooves become shallower, and the number of holes becomes lower. Meanwhile, the S4 surface is much flatter and smoother than the other three surfaces. The particles of the S4 surface are obviously refined, the cluster boundaries become narrower and blurred, and the number of holes and grooves reduces to the lowest number.

**Figure 3:**
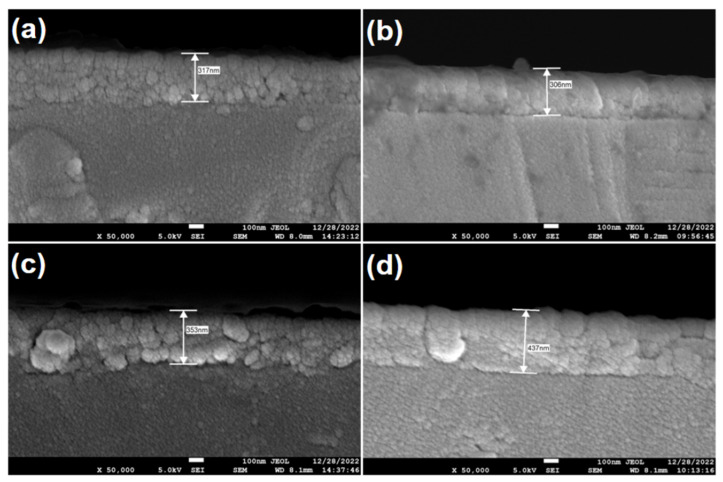
The cross-sectional SEM images of the MZNMAO films with different annealing temperatures: (**a**) 650 °C, (**b**) 750 °C, (**c**) 850 °C, (**d**) 950 °C.

In addition, the cross-sections of the S1~S4 films were characterized in order to measure the film thicknesses (see Figure 3). The thickness values of the four films are 317, 306, 353, and 437 nm.

**Figure 4:**
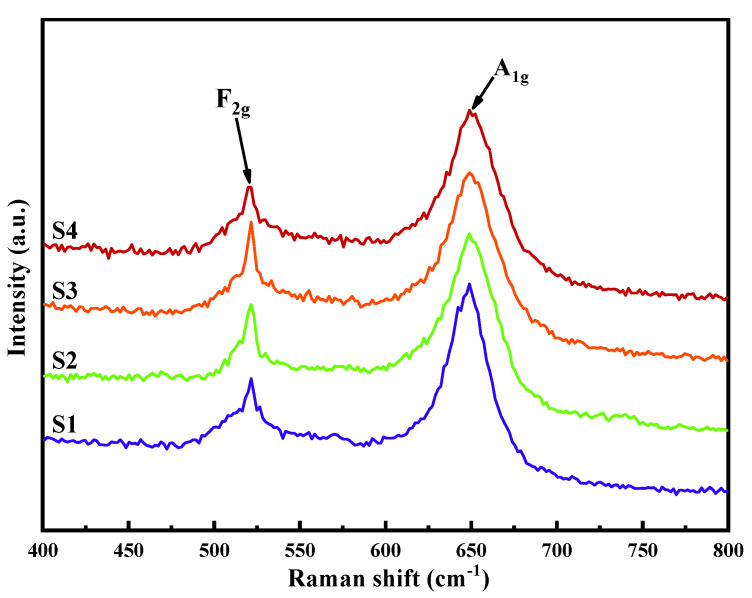
Raman spectra of the MZNMAO films with different annealing temperatures: S1 for 650 °C, S2 for 750 °C, S3 for 850 °C, and S4 for 950 °C.

The Raman spectra of the MZNMAO films with different annealing temperatures are shown in Figure 4. The peak at 521 cm^−1^ is attributed to the F_2g_ vibration mode of the films, which represents the symmetric bending vibration of the Mn^4+^-O^2-^ bonding in the O octahedron. The peak at 649 cm^−1^ is ascribed to the A_1g_ vibration mode of the films, which represents the symmetric telescoping vibration of the Mn^3+^-O^2-^ bonding in the O octahedron [11]. The relative change in the peak intensities represents the content change in the cations’ chemical bonding in the film. From the figure, the content of the Mn^3+^ (A1g peak) cation does not show obvious changes with the temperature. However, the content of the Mn^4+^ (F_2g_ peak) cation is relatively weak for the S1 curve, slightly increases in its intensity for S2, and maintains its intensity for S3 and S4. In order to further investigate the evolution of Mn cations with the temperatures, XPS spectra were tested.

**Figure 5:**
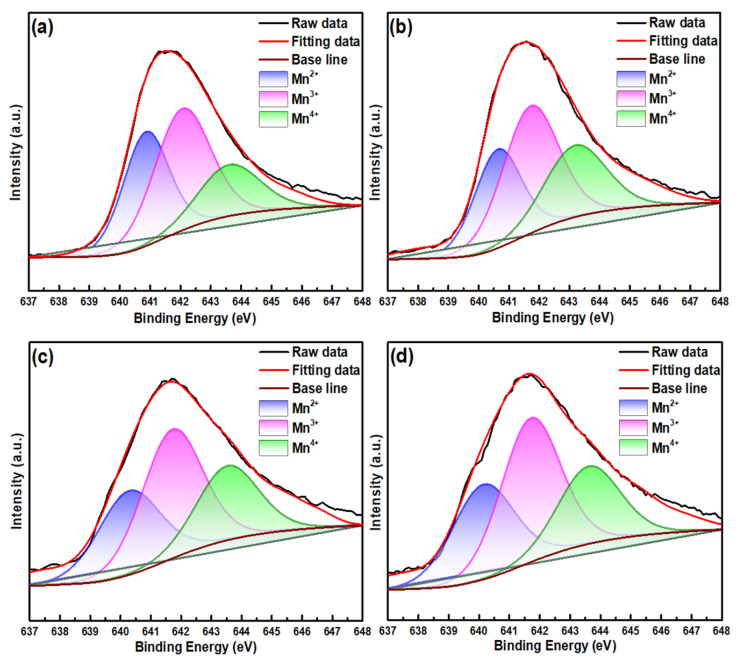
Mn 2p 3/2 XPS pattern of the MZNMAO films with different annealing temperatures: (**a**) 650 °C, (**b**) 750 °C, (**c**) 850 °C, (**d**) 950 °C.

The electrical properties of spinel-structured MZNMAO films are greatly affected by the valences and distribution of Mn cations [12]. The evolution of Mn^2+^, Mn^3+^, and Mn^4+^ cations with different annealing temperatures in MZNMAO films were investigated using the XPS test (see Figure 5).

Split-peak fitting of the Mn 2p3/2 mapping yields three characteristic sub-peaks of Mn^2+^, Mn^3+^, and Mn^4+^ with respective binding energies of 640.5, 641.8, and 643.5 eV. Calculating the integral of the relative areas of each sub-peak, the relative contents of manganese ions [13] are shown in Table 1. With the increase in the annealing temperature, the content of Mn^3+^ remains unchanged. The contents of Mn^2+^ and Mn^4+^ vary in the opposite trend: if the content of Mn^2+^ decreases, that of Mn^4+^ tends to increase, or vice versa. Meanwhile, the ratio of Mn^3+^/Mn^4+^ first decreases to the smallest value from S1 to S2 and then keeps slightly increasing from S2 to S4.

**Figure 6:**
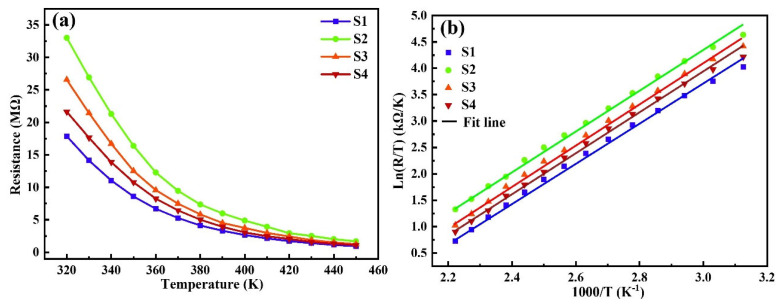
(**a**) Resistance–temperature curves and (**b**) Ln(R/T)−1000/T curves of the MZNMAO films with different annealing temperatures.

Figure 6a illustrates the relationship between the film resistance *R* and the annealing temperature *T*. The *R*-*T* curve is drawn according to the equation *R* = C*T*exp(*E*a/Kb*T*), where C is a constant, *E*a the activation energy, Kb the Boltzmann’s constant, and *T* the absolute temperature [14]. As the temperature increases, the *R* value of MZNMAO films decreases exponentially, indicating a typical negative thermal coefficient property. When the annealing temperature increases from S1 to S2, the film resistance substantially increases. However, from S2 to S4, the film resistance slightly decreases. The curves of Ln(*R*/*T*)−1000/*T* are fitted and shown in Figure 6b. The fitted curves exhibit linear relationships, suggesting that the conductive behaviors of the MZNMAO films are through small polaron hopping. The electrical parameters (see Table 2) of the films are calculated according to *B*_T1/T2_ = Ln (*R*_1_/*R*_2_)/(1/*T*_1_ − 1/*T*_2_), where *R*_1_ and *R*_2_ are the corresponding resistance values at *T*_1_ and *T*_2_. B_T1/T2_ is a material constant (the higher the value of *B*_T1/T2_ is, the higher the temperature sensitivity of the thermistor [15]). The value of *BT*_1_/*T*_2_ is calculated from the resistances measured at 320 (*T*_1_) and 420 (*T*_2_) K, denoted as *B*_320/420_. *E*a is calculated according to the equation of *E*a = *B*_320/420_ × Kb.

## 4. Conclusions

In this work, MZNMAO HEO films with different annealing temperatures were fabricated on Si substrates using electron beam evaporation plus the post-annealing in-air method. All the samples form spinel phases and exhibit excellent NTC properties over the entire test temperature range. When the annealing temperature increases, the crystalline quality of the spinel phase becomes better. Especially at 850 °C, a uniform spinel phase forms, while for films at lower or higher temperatures than 850 °C, there are impurity phases emerging, either due to incomplete inter-diffusion or precipitation. Considering that the film at 750 °C annealing has the best electrical properties, the impurity phase does not greatly affect the conductive behavior of MZNMAO HEO films.

## Figures and Tables

**Table 1:** Proportion of manganese ions in different valence states.

Sample	Mn^2+^	Mn^3+^	Mn^4+^	Mn^3+^/Mn^4+^
S1	34.3%	44.8%	20.8%	2.152
S2	28.6%	44.0%	27.4%	1.607
S3	29.5%	44.4%	26.1%	1.700
S4	30.6%	44.5%	24.9%	1.783

**Table 2:** Resistance, *B*-value, and activation energy of different samples *E*a.

Sample	R_320_ (MΩ)	R_420_ (MΩ)	B_320/420_ (K)	*E*a (eV)
S1	17.87	1.714	3150.7	0.2715
S2	33.01	2.939	3250.8	0.2801
S3	26.56	2.431	3213.6	0.2769
S4	21.63	2.042	3172.0	0.2733

## Data Availability

The original contributions presented in the study are included in the article; further inquiries can be directed to the corresponding author.
